# Effectiveness of simple tracing test as an objective evaluation of hand dexterity

**DOI:** 10.1038/s41598-019-46356-9

**Published:** 2019-07-09

**Authors:** Tomohiro Nishi, Kiyohiro Fukudome, Kazutaka Hata, Yutaka Kawaida, Kazunori Yone

**Affiliations:** 1 Graduate School of Health Sciences, Kagoshima University, Kagoshima, 890-8544 Japan; 2 School of Health Sciences, Faculty of Medicine, Kagoshima University, Kagoshima, 890-8544 Japan

**Keywords:** Spinal cord diseases, Disability

## Abstract

This study aimed to demonstrate that the simple tracing test (STT) is useful for assessing the hand dexterity in patients with cervical spondylotic myelopathy (CSM) by comparing STT scores between healthy volunteers and CSM patients. This study included 25 CSM patients and 38 healthy volunteers. In the STT, the participants traced a sine wave displayed on a tablet device at a comfortable pace, and the tracing accuracy, changes in the total sum of pen pressures, and tracing duration were assessed. Data were analyzed using an artificial neural networks (ANN) model to obtain STT scores. All participants were evaluated using the subsection for the upper extremity function of the Japanese Orthopaedic Association (JOA) scoring system for cervical myelopathy (JOA subscore for upper extremity function) and the grip and release test (GRT). The results were compared with the STT scores. The mean STT scores were 24.4 ± 32.8 in the CSM patients and 84.9 ± 31.3 in the healthy volunteers, showing a significant difference. The STT scores showed highly positive correlations with both the JOA subscore for upper extremity function (*r* = 0.66; *P* < 0.001) and GRT values (*r* = 0.74; *P* < 0.001). Furthermore, receiver operating characteristic analysis showed an area under the curve of 0.89 (95% confidence interval, 0.76–1.00), demonstrating that STT has excellent discriminative ability. This study revealed that STT enables accurate assessment of the hand dexterity in CSM patients.

## Introduction

Cervical spondylotic myelopathy (CSM) is a spinal cord disorder resulting from age-related degeneration of the cervical spine; its major symptoms include sensory abnormalities of the limbs, movement disorders, and bladder and rectal disturbances^1–3^. Myelopathy hand is a typical symptom and causes serious difficulty in daily life. While the subsection for the upper extremity function of the Japanese Orthopaedic Association scoring system for cervical myelopathy (JOA subscore for upper extremity function) is often used to assess the severity of myelopathy hand^4,5^, the JOA subscore for upper extremity function is based on subjective assessment by patients and has additional drawbacks^6–8^. For instance, the score is based on a 5-point scale from 0 to 4 and cannot reflect minute changes; moreover, some assessment items, such as the use of chopsticks, are not internationally applicable criteria. A commonly used objective assessment method is the grip and release test (GRT), in which subjects clench and unclench their hands as frequently as possible for 10 seconds and are evaluated according to the frequency of movements^1^. However, this test must be administered by investigators with a certain level of experience who can differentiate normal clenching and unclenching of the hand from trick motion, a compensatory movement^9^. Furthermore, it is also uncertain whether the speed of clenching and unclenching the hand can be an indicator of limitations in daily activities.

We developed the simple tracing test (STT) to quantitatively assess the hand dexterity. In this test, subjects trace a sine wave displayed on a tablet device at a comfortable pace, and the tracing accuracy, changes in the total sum of pen pressures, and tracing duration are analyzed. This study aimed to demonstrate that STT is useful for assessing the hand dexterity in CSM patients by comparing STT scores between healthy volunteers and CSM patients.

## Results

All participants performed and completed STT (there were no participants who did not begin the test or dropped out in the middle of the test). Through the learning process using the training data set, which included data on 30 participants, the artificial neural networks (ANN) model was optimized for estimating the probability of being a CSM patient. With this ANN model, STT scores of 33 participants were calculated from the input data of the validation data set. The mean STT scores were 24.4 ± 32.8 in the patient group and 84.9 ± 31.3 in the control group, showing a significant difference (*P* < 0.001).

The JOA subscore for upper extremity function of the 15 patients in the validation data set were 3 points in 10 patients, 2 points in 1 patient, and 1 point in 4 patients. Meanwhile, all scores of the 18 healthy volunteers were 4 points. The mean GRT values were 15.1 ± 5.9 times in the patient group and 29.0 ± 7.0 times in the control groups, showing a significant difference (*P* < 0.001). When the correlations between STT and JOA subscore for upper extremity function and between STT scores and GRT values were analyzed using the Spearman rank correlation coefficient, highly positive correlations were observed between STT and JOA subscore for upper extremity function (*r* = 0.66; *P* < 0.001) and between STT scores and GRT values (*r* = 0.74; *P* < 0.001).

**Figure 1:**
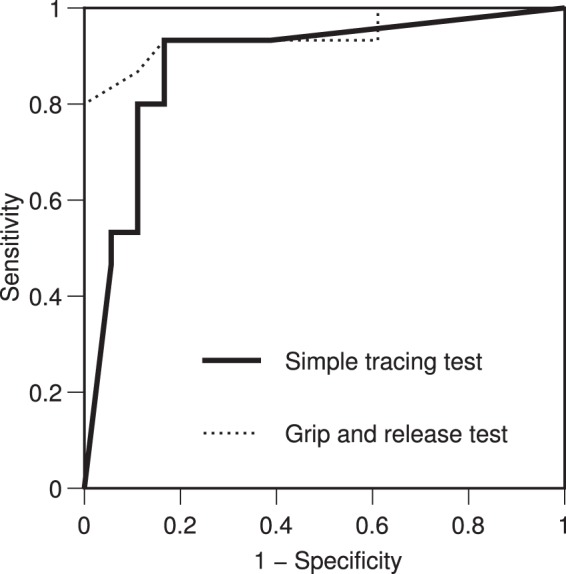
ROC curves for simple tracing test (STT) and GRT. The solid line shows STT and the dotted line shows GRT.

To assess the ability of STT to distinguish between patients and healthy volunteers, receiver operating characteristic (ROC) analysis was performed on STT scores as shown in Fig. 1. This analysis yielded a cutoff value of 84.4, a sensitivity of 0.93, a specificity of 0.83, and an area under the curve (AUC) of 0.89 (95% confidence interval [CI], 0.76–1.00) (Table 1). When the patient group was divided by the cutoff value, 14 patients were correctly diagnosed as having CSM, whereas only 1 patient was incorrectly diagnosed as not having CSM. In the control group, 15 healthy volunteers were correctly diagnosed as not having CSM, whereas 3 volunteers were incorrectly diagnosed as having CSM. Likewise, ROC analysis on GRT values yielded a cutoff value of 19.0, a sensitivity of 0.87, a specificity of 0.89, and an AUC of 0.95 (95% CI, 0.86–1.00). When the patient group was divided by this cutoff value in the same manner as STT scores, 13 patients were correctly diagnosed as having CSM, whereas 2 patients were incorrectly diagnosed as not having CSM. In the control group, 16 healthy volunteers were correctly diagnosed as not having CSM, whereas 2 volunteers were incorrectly diagnosed as having CSM. Of the participants who were incorrectly diagnosed using STT, 2 healthy volunteers and 1 patient were also incorrectly diagnosed using GRT.

**Table 1:** Sensitivity and specificity calculated by ROC analysis.

	Sensitivity	Specificity	AUC
Tracing accuracy	0.87	0.83	0.92
Total sum of pen pressures	0.67	0.50	0.58
Maximum change in pen pressures	0.73	0.72	0.75
Tracing duration	0.67	0.56	0.54
STT score	0.93	0.83	0.89
GRT score	0.87	0.89	0.95

ROC analysis was also performed on the input data (the tracing accuracy, total sum of pen pressures during tracing, maximum change in pen pressures during tracing, and tracing duration) to determine the sensitivity and specificity. The sensitivity and specificity were 0.87 and 0.83 for the tracing accuracy, 0.67 and 0.50 for the total sum of pen pressures during tracing, 0.73 and 0.72 for the maximum change in pen pressures during tracing, and 0.67 and 0.56 for the tracing duration, respectively. None of the variables showed better sensitivity or specificity than STT scores (Table 1).

The intra-observer reliability of STT score was high, intra-class correlation coefficient 0.78 (95% CI, 0.66–0.88). As shown in Table 2, the scores for each cycle were higher in the control group than in the patient group at any cycle.

**Table 2:** Scores for each cycle.

	Patients	Controls
First cycle	23.0 ± 23.2	88.4 ± 10.8
Second cycle	26.8 ± 19.5	82.8 ± 26.8
Third cycle	20.1 ± 26.1	75.8 ± 28.4

## Discussion

Although, myelopathy hand is a typical symptom of CSM, it is difficult to assess the hand dexterity of this symptom. To solve these problems, we developed STT. In this test, the use of a pen, which is a daily activity, is assessed. Specifically, subjects trace a sine wave displayed on a tablet device at a comfortable pace, and the tracing accuracy, changes in the total sum of pen pressures, and tracing duration are analyzed. Because a commercially available personal computer or pen display tablet is used, this test can be performed at low cost and with simple structure. The analysis process is automated by using ANN, so that the subjective viewpoint of investigators does not affect results. Because the start and end of the test are automatically determined by a computer, investigators are free of the need to simultaneously perform multiple tasks, such as timing and counting^10^. In addition, because STT does not require differentiation of normal clenching and unclenching of the hand from trick motion, investigators can easily use the test regardless of their levels of experience.

Hand dominancy and disease might have influenced the results. In our preparatory experiment, we confirmed that hand dominance exerted a strong influence on tracing in both healthy volunteers and patients with cervical myelopathy. If a patient with cervical myelopathy were to use the non-dominant hand in testing, it would be difficult to determine whether the effects were due to that non-dominant hand use or, rather, to the disease. Therefore, in this study, only dominant hands were used. Although non-dominant hands were not included in this study, we regarded the evaluation as being useful considering that chopsticks are usually held with the dominant hand. In addition, it was examined whether three drawing tasks, i.e., a straight line, a sawtooth wave, and the sine wave were suitable for testing hand dexterity. We found that for these three drawing tasks, to complete any one of them, the examinees needs the gross motor skills for these drawing tasks and, moreover, the fine motor skills for a sine-wave drawing task.

Because there were no participants who did not begin STT or discontinued it in the middle of this study, the STT tracing task appears to have been easy for the participants to perform. The 4 variables (the tracing accuracy, total sum of pen pressures during tracing, maximum change in pen pressures during tracing, and tracing duration) calculated from STT results can be used separately for assessment. In fact, the sensitivity and specificity were high at 0.87 and 0.83, respectively, for the tracing accuracy and 0.73 and 0.72, respectively, for the maximum change in pen pressures during tracing. However, by comprehensively assessing the 4 variables through the ANN model, the sensitivity of STT was improved to 0.93, although its specificity was the same as that of the tracing accuracy (0.83). Because the ANN model can characteristically reveal the hidden association between input and output data^11–13^, this excellent feature might have contributed to the improved sensitivity in analysis of STT results. STT scores, which are derived from analysis of STT results, can be used to distinguish CSM patients and healthy volunteers. The high diagnostic capability of the score was demonstrated by an AUC of 0.89 (95% CI, 0.76–1.00) in ROC analysis. Moreover, the STT score was highly correlated with the JOA subscores for upper extremity function (*r* = 0.66; *P* < 0.001) and GRT values (*r* = 0.74; *P* < 0.001), supporting the high validity of STT. Furthermore, intra-observer intra-class correlation coefficient of 0.78 was demonstrated the high reliability of the STT. The sensitivity of STT scores yielded by ROC analysis was high and exceeded that of GRT values. This suggests that STT can be used for upper limb function screening for CSM.

STT is so simple that subjects can perform the task at their own pace. Moreover, despite the small size of the validation data set, which included data on 30 participants comprising 10 patients and 20 healthy volunteers, the accuracy of differentiation was high. In STT, the ANN model can perform assessment through prior learning with the training data set including data on CSM patients and healthy volunteers. Because the ANN model itself is not specific for CSM, the model can be expected to be applicable to not only CSM but also other diseases causing motor dysfunction of the upper limbs, if training data sets are prepared for patients with targeted diseases, such as cervical spinal cord tumor.

Conventional functional tests are considered to be affected by not only the hand dexterity of motor dysfunction but also age-related impairment in motor function^6^. In this study, it was difficult to determine the presence of such effects because of inclusion of participants within a limited range of ages. Furthermore, the test results may be affected by cognitive function, visual acuity, etc. Further studies may be needed to investigate these effects. In addition, the reproducibility of STT should also be demonstrated in a future study. In general, performance assessment requires an enormous amount of time and cost^7^. However, STT does not require any expensive devices or special measurement environments, while a series of operations from collection of data necessary for assessment to output of results can be completed in approximately 1 minute. This is desirable for not only investigators but also subjects.

This study demonstrated that the STT score correlated to the JOA subscore for upper extremity function and can distinguish between CSM patients and healthy volunteers. When using not only STT score but also the remaining factor such as age and disease duration, we may even measure severity of CSM based on ANN method.

## Methods

### Ethical considerations

This study was conducted in accordance with the World Medical Association Declaration of Helsinki and was approved by the ethics committees of Faculty of Medicine and University Hospital, Kagoshima University. All patients were fully, informed about the study content and gave their written consent.

### Patients

This study included 25 patients who were diagnosed with CSM based on somatic symptoms and imaging findings by specialists. All patients were right-handed and complained of numbness or stiffness of their hands. The data on tracing task performance of the first 10 patients were used for training of the ANN model, whereas those of the subsequent 15 patients were used for validation of its predictive ability.

### Controls

The control group consisted of 38 healthy volunteers aged 20–29 years or 60 years or older, who had not undergone brain or spinal surgery, had no history of brain or neurological diseases, and were free of symptoms associated with sensory or movement disorders (e.g., numbness, clumsiness, and motor weakness)^6^. All volunteers were right-handed. The data on tracing task performance of the first 20 volunteers were used for training of the ANN model, whereas those of the subsequent 18 volunteers were used for validation of its predictive ability.

### Procedure

**Figure 2:**
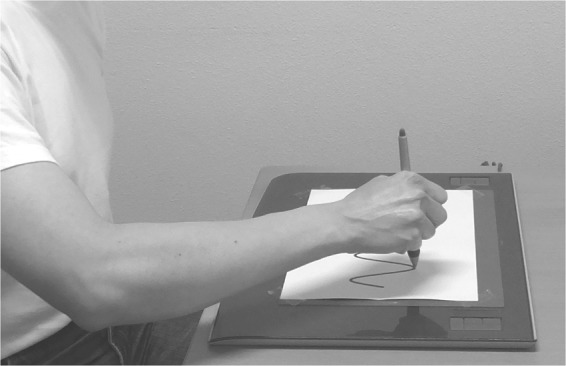
Simple tracing test. Subjects began tracing from the left side toward right side at a comfortable pace. They were instructed not to touch the device with their hands, their fingers, or anything except the tip of the pen while performing the tracing task.

**Figure 3:**
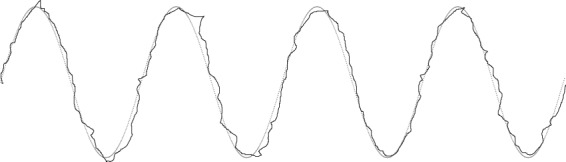
Digitized traced line. The solid line shows the trajectory of the pen tip moved by a CSM patient during the test. The dotted line shows original, sine-wave curve. This patient was judged as a CSM patient in both STT and GRT.

Motor function of the hands and fingers of the participants was examined by 2 common methods, i.e., the JOA subscore for upper extremity function (Table 3) and GRT, in which the participants clenched and unclenched their hands as frequently as possible for 10 seconds and were evaluated according to the frequency of the movements. STT was then administered (Fig. 2). A task figure printed on a sheet of paper was attached to the pen tablet surface, and the subjects were asked to trace the figure one time and from the left side toward the right side as accurately as possible at their own pace. The figure used for the task was a 4-cycle sine wave with an amplitude of 35 mm and a wavelength of 62 mm. From digitized traced lines (Fig. 3), the following 4 variables were obtained: the tracing accuracy, total sum of pen pressures during tracing, maximum change in pen pressures during tracing, and tracing duration. In STT, we covered an interval of 3 cycles in which the first and last half cycles were excluded. For tracing accuracy, the total sum of the distance between the traced figure and the task figure from the start to end of STT was calculated. As the traced figure deviated from the task figure, this variable increased. For the total sum of pen pressures during tracing, the total sum of changes in pen pressure from the start to the end of STT was calculated. When the participants could not trace the figure smoothly, this variable increased. The maximum change in pen pressure during tracing was defined as the largest change observed in pen pressure between the start and end of STT. When the pen was detached from the display screen during tracing, a sudden change in pen pressure was recorded. The tracing duration was the time required to perform the task from the start to end.

**Table 3:** Subsection for the upper extremity function of Japanese Orthopaedic Association Scoring System for Cervical Myelopathy^4^.

Point	Statement
0	Unable to feed oneself with any tableware including chopsticks, spoon, or fork, and/or unable to fasten buttons of any size
1	Can manage to feed oneself with a spoon and/or fork but not with chopsticks
2	Either chopstick-feeding or writing is possible but not practical, and/or large buttons can be fastened
3	Either chopstick-feeding or writing is clumsy but practical, and/or cuff buttons can be fastened
4	Normal

### Assessment using STT

To generate data sets, the input data including the 4 variables calculated from STT results (the tracing accuracy, total sum of pen pressures during tracing, maximum change in pen pressures during tracing, and tracing duration) and the output data for the attributes of the participants (CSM or not) were collected as a pair and divided into a training data set and a validation data set, according to the order of enrollment. The training data set (see Supplementary Table S1), which included data on 10 patients and 20 healthy volunteers, was used only for developing an optimal ANN model. The validation data set (see Supplementary Table S2), which included data on 15 patients and 18 healthy volunteers, was used to validate the predictive ability of the ANN model for clinical data.

**Figure 4:**
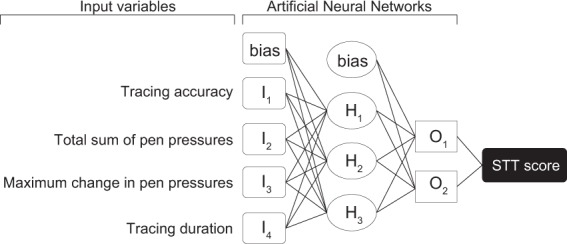
STT score yielded by the system using three layer perceptron ANN. When the 4 variables calculated from STT results were entered, the system yielded a “probability of being likely to be a healthy individual (STT score)”. Hyperbolic tangent function was chosen as activation function of Hidden layer.

ANN is a theoretical framework of information processing that mimics the human brain^13,14^. It is used for data that cannot be processed by conventional statistical methods and can elucidate hidden associations between input and output data^11–13,15,16^. A multilayer perceptron, a common ANN model, can yield appropriate answers through a learning process, similar to how experienced physicians make an accurate diagnosis based on accumulated experiences^17^. To analyze STT results, we used a 3-layer perceptron and a training algorithm of backpropagation to develop a system to predict whether the participants met the criteria for CSM based on data from the tracing task performance^18^. Specifically, as shown in Fig. 4, when the 4 variables calculated from STT results were entered, the system yielded a “probability of being likely to be a healthy individual.” In this study, the output of this system was defined as the STT score. The STT score was a variable with values ranging from a minimum of 0 to a maximum of 100. Scores closer to 0 indicated that participants were likely to have CSM, whereas scores closer to 100 indicated that participants were unlikely to have CSM. Through the learning process using the training data set, which included the data on 30 participants (10 patients and 20 healthy volunteers), the ANN model was adjusted to yield optimal outputs (Table 4).

**Table 4:** Parameter estimates.

Predictor		Predicted				
		Hidden Layer			Output Layer	
		H_1_	H_2_	H_3_	O_1_	O_2_
Input Layer	Bias	−0.805	0.609	1.315		
	I_1_	−0.089	1.922	4.643		
	I_2_	0.720	−1.611	−3.705		
	I_3_	0.000	−0.469	−0.599		
	I_4_	1.378	−2.402	−4.015		
Hidden Layer	Bias				0.497	0.500
	H_1_				−0.641	0.640
	H_2_				−1.235	1.220
	H_3_				1.123	−1.120

Next, the predictive ability of the ANN model was validated with the validation data set, which included data on 33 participants (15 patients and 18 healthy volunteers). In the validation process, the input data of the validation data set were first entered into the ANN model to obtain STT scores. Then, these scores were compared with the output data of the validation data set. The border STT score between the healthy volunteers and patients was determined using ROC analysis^19^.

### Data Analysis

In this study, the ability to distinguish between the patients and healthy volunteers was assessed in terms of sensitivity and specificity^17^. Sensitivity was defined as the proportion of participants who were accurately identified as patients by the cutoff value in the patient group. Specificity was defined as the proportion of participants who were accurately identified as healthy volunteers by the cutoff value in the control group. The cutoff value was defined as the lowest value derived from the formula, (1 − sensitivity)^2^ + (1 − specificity)^2^, on the ROC curve. ROC analyses were performed separately for the input variables, STT scores, and GRT values to calculate cutoff values, sensitivity, specificity, and AUC. An unpaired t-test was used to compare mean values between the patient and control groups. The correlations between STT scores and JOA subscores for upper extremity function and between STT scores and GRT values were assessed with the Spearman rank correlation coefficient. In this study, a *P* value less than 0.05 was considered to indicate significance. All analyses were performed with IBM SPSS Statistics 21 (IBM, Armonk, NY, USA).

### Verification of intra-observer reliability

From the original tracing data, one-cycle segments were abstracted individually to create a first, second and third cycle data. There were 90 (30 × 3) one-cycle segments data for training and 99 (33 × 3) for validation. Similar to original tracing data, after the learning process using 90 training data set, we calculated the scores of 99 validation data sets. The intra-observer reliability of the STT was examined by determining the intra-class correlation coefficient using these data.

## Supplementary information

The training and validation data sets

## References

[1] Ono K (1987). Myelopathy hand. New clinical signs of cervical cord damage. J. Bone Joint Surg. Br..

[2] Young, W. F. Cervical spondylotic myelopathy: a common cause of spinal cord dysfunction in older persons. *Am*. *Fam*. *Physician* **62**, 1064–1070, 1073 (2000). 10997531

[3] Yukawa Y (2013). Quantifiable tests for cervical myelopathy; 10-s grip and release test and 10-s step test: standard values and aging variation from 1230 healthy volunteers. J. Orthop. Sci..

[4] Yonenobu K, Abumi K, Nagata K, Taketomi E, Ueyama K (2001). Interobserver and intraobserver reliability of the Japanese Orthopaedic Association scoring system for evaluation of cervical compression myelopathy. Spine.

[5] Holly LT (2009). Functional outcomes assessment for cervical degenerative disease. J. Neurosurg. Spine.

[6] Yukawa Y (2009). “Ten Second Step Test” as a New Quantifiable Parameter of Cervical Myelopathy. Spine.

[7] Mihara H (2010). A New Performance Test for Cervical Myelopathy The Triangle Step Test. Spine.

[8] Numasawa T (2012). Simple Foot Tapping Test as a Quantitative Objective Assessment of Cervical Myelopathy. Spine.

[9] Hosono N (2008). A simple performance test for quantifying the severity of cervical myelopathy. J. Bone Joint Surg. Br..

[10] Hosono N (2012). Postoperative 24-Hour Result of 15-Second Grip-and-Release Test Correlates With Surgical Outcome of Cervical Compression Myelopathy. Spine.

[11] Baxt WG (1995). Application of artificial neural networks to clinical medicine. Lancet.

[12] Cacciafesta M (2001). Neural network analysis in predicting 2-year survival in elderly people: a new statistical-mathematical approach. Arch. Gerontol. Geriatr..

[13] Kupusinac A, Doroslovacki R, Malbaski D, Srdic B, Stokic E (2013). A primary estimation of the cardiometabolic risk by using artificial neural networks. Comput. Biol. Med..

[14] Spelt L, Nilsson J, Andersson R, Andersson B (2013). Artificial neural networks–a method for prediction of survival following liver resection for colorectal cancer metastases. Eur. J. Surg. Oncol..

[15] Dybowski R, Weller P, Chang R, Gant V (1996). Prediction of outcome in critically ill patients using artificial neural network synthesised by genetic algorithm. Lancet.

[16] Azar AT (2013). Fast neural network learning algorithms for medical applications. Neural Compt Appl.

[17] Cross SS, Harrison RF, Kennedy RL (1995). Introduction to neural networks. Lancet.

[18] Yardimci A (2009). Soft computing in medicine. Appl Soft Comput.

[19] Meistrell ML (1990). Evaluation of neural network performance by receiver operating characteristic (ROC) analysis: examples from the biotechnology domain. Comput. Methods Programs Biomed..

