# Supplementary material for: Effectiveness of simple tracing test as an objective evaluation of hand dexterity
Source: Sci Rep. 2019 Jul 9;9:9915. doi: 10.1038/s41598-019-46356-9 (PMC6616365; doi:10.1038/s41598-019-46356-9)
Supplement: Supplementary file 1 — The training and validation data sets [file 41598_2019_46356_MOESM1_ESM.docx]

**Effectiveness of simple tracing test as an objective evaluation of hand dexterity**

Tomohiro Nishi^1,*^, Kiyohiro Fukudome^2^, Kazutaka Hata^1^, Yutaka Kawaida^1^ and Kazunori Yone^2^

^1^Graduate School of Health Sciences, Kagoshima University, Kagoshima, 890-8544, Japan

^2^School of Health Sciences, Faculty of Medicine, Kagoshima University, Kagoshima, 890-8544, Japan

**Supplementary Table S1.** The training data set.

| Input variables | | | |  | Output |
| --- | --- | --- | --- | --- | --- |
| Tracing accuracy  (m) | Total sum of pen pressures  (kgf) | Maximum change in pen pressures  (kgf) | Tracing duration (s) |  |  |
| 3.82  4.46  11.91  3.48  4.78  6.21  4.32  15.76  3.13  3.05 | 4.58  12.74  7.17  1.30  1.88  4.24  1.09  0.91  1.62  2.02 | 0.0552  0.0672  0.0488  0.0135  0.0584  0.0498  0.0131  0.0062  0.0731  0.0226 | 14.72  23.78  33.50  10.01  11.51  29.37  10.52  29.28  25.94  12.11 |  | CSM  CSM  CSM  CSM  CSM  CSM  CSM  CSM  CSM  CSM |
| 3.58  1.81  2.12  5.42  2.82  1.70  4.71  2.13  3.38  8.68 | 0.84  1.17  1.80  2.15  1.48  2.71  8.72  4.99  1.87  4.04 | 0.0099  0.0234  0.0159  0.0125  0.0219  0.0139  0.0258  0.0184  0.0136  0.0091 | 11.43  12.36  10.84  24.27  9.25  13.73  22.00  21.23  20.72  29.74 |  | Normal  Normal  Normal  Normal  Normal  Normal  Normal  Normal  Normal  Normal |
| 2.59  3.60  3.76  2.95  2.93  2.85  2.20  2.07  1.86  1.37 | 2.32  2.79  2.76  7.38  3.72  5.72  2.75  1.84  0.75  0.61 | 0.0132  0.0106  0.0107  0.0151  0.0105  0.0114  0.0084  0.0099  0.0066  0.0080 | 21.43  25.84  33.80  27.75  28.67  44.71  30.99  13.49  7.84  6.16 |  | Normal  Normal  Normal  Normal  Normal  Normal  Normal  Normal  Normal  Normal |

**Supplementary Table S2.** The validation data set.

| Input variables | | | |  | STT score | GRT | JOA subscore for upper extremity function |
| --- | --- | --- | --- | --- | --- | --- | --- |
| Tracing accuracy  (m) | Total sum of pen pressures  (kgf) | Maximum change in pen pressures  (kgf) | Tracing duration  (s) |  |  |  |  |
| 3.52  12.83  5.80  8.63  4.68  16.17  3.76  9.96  6.71  6.95  16.37  6.70  12.12  5.01  8.88 | 1.53  3.05  2.41  6.98  1.09  6.72  0.77  1.73  2.13  3.46  4.72  2.04  4.61  1.85  2.05 | 0.0385  0.0107  0.0282  0.0504  0.0160  0.0173  0.0070  0.0139  0.1274  0.0155  0.0256  0.0411  0.0336  0.0175  0.0118 | 8.39  29.65  13.30  29.47  17.99  40.18  8.33  20.87  8.19  19.68  21.95  23.10  33.50  15.60  27.05 |  | 42  0  54  0  100  0  0  38  0  21  0  8  0  20  83 | 15  11  18  11  20  32  17  11  14  9  7  17  14  15  15 | 3  3  3  3  3  3  3  1  1  1  1  3  3  3  2 |
| 4.38  7.03  6.44  4.01  4.41  4.94  3.26  2.29 | 6.55  4.78  2.91  3.82  2.03  10.43  2.35  1.38 | 0.0951  0.0342  0.0218  0.0133  0.0067  0.0186  0.0081  0.0083 | 30.71  54.71  22.44  22.16  30.19  42.50  14.73  8.32 |  | 0  15  43  100  100  91  100  95 | 18  18  22  35  30  21  23  20 | 4  4  4  4  4  4  4  4 |
| 2.16  3.51  1.92  1.84  2.70  2.97  2.00  1.99  1.80  2.60 | 2.09  1.91  0.60  0.82  2.56  0.93  0.66  1.03  0.89  5.47 | 0.0123  0.0118  0.0047  0.0048  0.0142  0.0043  0.0096  0.0058  0.0052  0.0331 | 20.98  12.34  10.90  11.89  15.62  17.26  8.19  8.85  15.21  23.45 |  | 100  85  100  100  100  100  99  100  100  100 | 30  33  30  36  35  31  38  29  34  39 | 4  4  4  4  4  4  4  4  4  4 |
